# Group A Rotavirus Associated with Encephalitis in Red Fox

**DOI:** 10.3201/eid2309.170158

**Published:** 2017-09

**Authors:** Chiara Busi, Vito Martella, Alice Papetti, Cristiano Sabelli, Davide Lelli, G. Loris Alborali, Lucia Gibelli, Daniela Gelmetti, Antonio Lavazza, Paolo Cordioli, M. Beatrice Boniotti

**Affiliations:** Istituto Zooprofilattico Sperimentale della Lombardia e dell’Emilia Romagna “Bruno Ubertini,” Brescia, Italy (C. Busi, A. Papetti, C. Sabelli, D. Lelli, G.L. Alborali, L. Gibelli, D. Gelmetti, A. Lavazza, P. Cordioli, M.B. Boniotti);; Università degli Studi di Bari, Bari, Italy (V. Martella)

**Keywords:** rotavirus, group A rotavirus, fox, brain, neurologic disease, viruses, Italy, meningitis/encephalitis

## Abstract

In 2011, a group A rotavirus was isolated from the brain of a fox with encephalitis and neurologic signs, detected by rabies surveillance in Italy. Intracerebral inoculation of fox brain homogenates into mice was fatal. Genome sequencing revealed a heterologous rotavirus of avian origin, which could provide a model for investigating rotavirus neurovirulence.

Group A rotaviruses are a major cause of diarrhea in humans and animals. Although group A rotaviruses infect particular species preferentially (homologous infection), they less frequently affect other species of mammals (heterologous infection), naturally and experimentally (*1*). In addition, there is evidence, albeit rare, that transmission of group A rotaviruses may occur between species of mammals and birds under natural and experimental conditions (*2*–*4*).

Group A rotaviruses have limited tissue tropism; infection is primarily restricted to cells of the small intestine. However, heterologous infection of mice with the rhesus group A rotavirus strain MMU 18006 was associated with extramucosal spread and hepatitis, but infections with bovine group A rotavirus WC3 and the homologous murine group A rotavirus EDIM were not (*5*), suggesting that some group A rotavirus strains may have unique or unexpected biological properties. In humans, group A rotavirus infection has been associated with acute encephalitis, although this association is based only on observational findings (*6*–*9*).

We detected a group A rotavirus strain in the brain of a fox with neurologic disorders. To determine the derivation of the virus, we further examined its genomic and biological features.

## The Study

In 2011, as part of Italy’s national surveillance program for rabies in wildlife, an adult red fox (*Vulpes vulpes*) with neurologic signs was captured. Because its general condition worsened, the animal was euthanized and screened for a panel of neuropathogens (Technical Appendix). Test results indicated that the animal was negative for rabies, canine distemper, Aujeszky’s disease, leishmaniasis, and flavivirus infection (online Technical Appendix). Following the standard diagnostic procedures for rabies, we inoculated brain homogenate from the fox intracerebrally into suckling and weanling mice. The suckling mice died after 3–4 days and the weanling mice after 5 days. However, immunofluorescence testing of the brains of all mice, using rabies-specific hyperimmune serum, produced negative results (data not shown).

**Figure 1:**
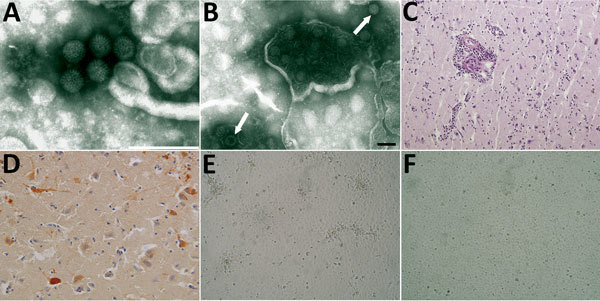
Images of brain of fox with group A rotavirus infection and brains of suckling and weanling mice inoculated with fox brain homogenates. A, B) Negative-staining electron microscopy. Presence of virions morphologically related to family *Reoviridae* from fox (A) and mouse (B) brain (arrows). Scale bar in panel A indicates 200 nm; in panel B, 100 nm. C, D) Histologic and immunohistochemical appearance of the cerebral cortex of the fox. C) Perivascular cuffing of inflammatory cells in the brain stained by hematoxylin and eosin (original magnification ×10). D) Viral antigen in the cytoplasm of neurons (immunohistochemistry, original magnification ×20). E) Foci with rounding cells of the confluent monolayers of Marc-145 cells infected with the brain homogenate from mouse at 2 days after inoculation (original magnification ×40). F) Mock cells (original magnification ×40).

Use of negative-staining electron microscopy revealed rotavirus-like virions in the fox and mouse brains (Figure 1, panels A, B). Histologically, several alterations/lesions, suggestive of acute inflammation, were observed in the cerebral cortex of the fox. Histologic analysis of gray matter revealed nonsuppurative encephalitis characterized by multifocal perivascular cuffing of lymphocytes, macrophages, and a few plasma cells as well as presence of multifocal small glial nodules. Perivascular accumulations varied from 1-cell thickness to thin cell accumulations (Figure 1, panel C). Neutrophils were observed within the lumen of some blood vessels and scattered in the gray matter. Neuronal necrosis and satellitosis were also present. By immunohistochemistry performed with a polyclonal serum raised against group A rotavirus, rotavirus antigen was detected in the cytoplasm of neurons, in dendrites, and in glial cells within inflamed areas of the brain (Figure 1, panel D).

An isolate, hereafter called fox-288356, was made from homogenates of the fox brain and from the brains of inoculated suckling and weanling mice, by using confluent monolayers of Marc-145 cells with and without trypsin. Cytopathic effect was characterized by foci of rounded cells, which tended to aggregate linearly on the surface of the monolayer and were clearly visible after 2 days (Figure 1, panel E). Electron microscopic observation identified rotavirus-like particles in the cell cryolysates (data not shown). The electropherotype of the cultured virus revealed a segmented genome characterized by a 5-1-3-2 profile with co-migration of segments 10 and 11 (Technical Appendix Figure 1).

**Figure 2:**
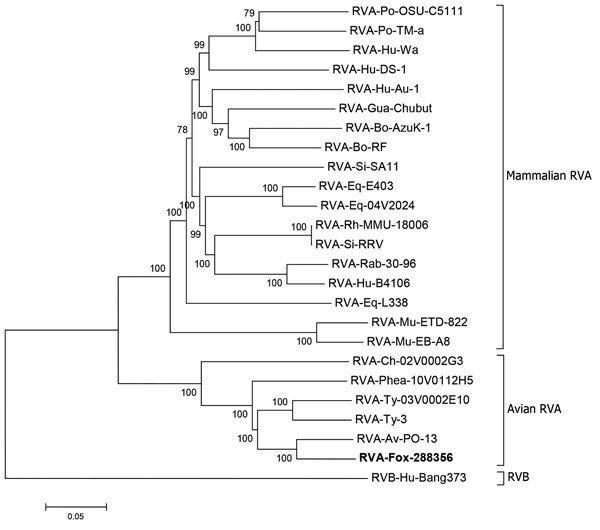
Phylogenetic analysis of RVA strain fox-288356. Analysis was performed on the basis of the concatenated nucleotide sequences of genomic segments. Fox- 288356 is correlated with RVA PO-13 (from pigeon) and clustered with the avian RVA. Reference sequences are identified by strain name and GenBank accession number. Scale bar indicates nucleotide substitutions per site. RVA, group A rotavirus; RVB, group B rotavirus.

The genome of fox-288356 was 18,849 nt and showed high sequence homology to avian strain PO-13, isolated from a pigeon (*2**,**4*). Homology was apparent in most genome segments (89%–94% nt and 91%–98% aa), except for the ninth segment, coding for viral protein (VP) 7 (86% nt and 88% aa), and the tenth segment, coding for nonstructural protein (NSP) 4 (79% nt and 83% aa) (Table 1). After phylogenetic analysis of the concatenated genome (Figure 2) and individual genome segments (Technical Appendix Figure 3), fox-288356 grouped with avian group A rotaviruses. The genomic constellation of fox-288356 was G18P[17]-R4-C4-M4-A4-I4-T4-N4-E19-H4 (Table 2).

**Table 1:** Comparison of genome segment sizes and sequence similarities among group A rotaviruses isolated from fox and pigeon*

Segment number/encoded protein	Nucleotide/amino acid length (genotype)		% Nucleotide sequence identity	% Amino acid sequence identity
	Fox-288356†	PO-13‡		
1/VP1	3,305/1,089 (R4)	3,302/1,088 (R4)	93	98
2/VP2	2,738/897 (C4)	2,738/897 (C4)	91	97
3/VP3	2,583/829 (M4)	2,583/829 (M4)	91	94
4/VP4	2,349/770 (P[17])	2,349/770 (P[17])	92)	95
5/NSP1	1,871/576 (A4)	1,870/576 (A4)	86	91
6/VP6	1,348/397 (I4)	1,348/397 (I4)	94	98
7/NSP3	1,092/306 (T4)	1,092/306 (T4)	89	95
8/NSP2	1,043/315 (A4)	1,042/315 (N4)	93	95
9/VP7	1,065/329 (G18)	1,065/329 (G18)	86	88
10/NSP4	726/169 (E19)	727/169 (E4)	77	83
11/NSP5	729/218 (H4)	729/218 (H4)	93	90

*NSP, nonstructural protein; VP, viral protein.  †Rotavirus isolated from a red fox. ‡Rotavirus isolated from a pigeon.

**Table 2:** Genomic constellation of avian group A rotavirus strains*

Group A rotavirus strain	VP7	VP4	VP6	VP1	VP2	VP3	NSP1	NSP2	NSP3	NSP4	NSP5
Fox-wt/ITA/288356/2011/G18P[17]	G18	P[17]	I4	R4	C4	M4	A4	N4	T4	E19	H4
Pigeon-tc/JPN/PO-13/1983/G18P[17]	G18	P[17]	I4	R4	C4	M4	A4	N4	T4	E4	H4
Bovine-wt/GER/993_83/1983/G18P[17]	G18	P[17]	I4								
Turkey-tc/GER/03V0002E10/2003/G22P[35]	G22	P[35]	I4	R4	C4	M4	A16	N4	T4	E11	H4
Group A rotavirus/tTurkey-tc/IRL/Ty-3/1979/G7P[35]	G7	P[35]	I4	R4	C4	M4	A16	N4	T4	E11	H14
Group A rotavirus/Turkey-tc/IRL/Ty-1/1979/G17P[38]	G17	P[38]	I4	R4	C4	M4	A16	N4	T4	E4	H4
Pheasant-tc/GER/10V0112H5/2010/G23P[37]	G23	P[37]	I4	R4	C4	M4	A16	N10	T4	E4	H4
Chicken-tc/GER/02V0002G3/2002/G19P[30]	G19	P[30]	I11	R6	C6	M7	A16	N6	T8	E10	H8

*Gray shading indicates homology**.** NSP, nonstructural protein; VP, viral protein.

Several amino acid mutations were present in the major antigenic regions (A, B, and C) of VP7 (Technical Appendix Figure 4) and in key residues of VP4 (Technical Appendix Figure 5). VP4 contained only 1 of the 3 arginine residues required for trypsin-mediated cleavage into the VP8* and VP5* subunits (*10*). This finding seems consistent with the ability of fox-288356 to grow in cell cultures in the absence of trypsin, a feature that has been observed for some avian group A rotaviruses (*11*).

We classified the NSP4 of fox-288356 as a novel E genotype, E19, as indicated by the Rotavirus Classification Working Group. We also found differences between the NSP4 of fox-288356 and other group A rotaviruses (Technical Appendix Figure 6).

## Conclusion

Although in humans group A rotaviruses are mainly associated with gastroenteritis, the literature indicates that group A rotaviruses may also be associated with acute encephalitis or encephalopathy (*6**–**9*). This correlation has been supposed for children in whom neurologic signs develop concomitantly or shortly after acute gastroenteritis caused by group A rotavirus (*7*,*9*) and after detection of group A rotavirus RNA in the cerebrospinal fluid of patients with neurologic signs (*6*,*8*). It remains unclear whether systemic spread of group A rotavirus and localization in the central nervous system is the result of host-related factors, whether it depends on intrinsic biological features of group A rotavirus strains, or whether it eventually results from a combination of both elements. Fox-288356 was probably responsible for the neurologic disease observed in the fox, as suggested by the results of our diagnostic investigations and by the inflammatory lesions in the brain of the animal.

Genomic characterization indicated that fox-288356 shared the same genetic backbone as avian strain PO-13 and avian-like bovine strain 993-83 (*2*). Under experimental conditions, oral inoculation of mice with pigeon group A rotavirus strain PO-13 infected and caused diarrhea in the mice, but inoculation with turkey group A rotavirus strain Tyr-1 did not (*4*). Also, the synthetic NSP4 toxic peptide of strain PO-13 elicited diarrhea in suckling mice (*12*). It is tempting to speculate that some avian group A rotaviruses (e.g.; group A rotaviruses with the PO-13 genome backbone) have the ability to cross the host-species barrier more easily than other avian group A rotaviruses (Table 1). Another bovine group A rotavirus strain, N2342, with a VP4 gene related to the avian strain PO-13, has been recently identified in Japan (*3*).

The virus isolated from the fox displayed a unique NSP4, which was proposed as a novel genotype, E19. NSP4 serves as an intracellular receptor for immature particles and interacts with viral capsid proteins during viral morphogenesis (*13*). NSP4 also acts as a viral enterotoxin (*13*,*14*), and the enterotoxic activity has been mapped to a region, the toxic peptide, spanning amino acids 114–135 of NSP4 (*14*). Changes in residues within the NSP4 toxic peptide have been associated with alterations in the toxigenic activity of NSP4 and in rotavirus virulence (*15*).

The detection of fox-288356 in the brain of a fox supports the accumulating clinical evidence for the association between group A rotaviruses and neurologic signs in human patients. Whether some group A rotavirus strains intrinsically possess the ability to spread to the central nervous system, thereby causing neurologic disease, remains to be explored.

Technical Appendix Additional methods used in study of group A rotavirus associated with encephalitis in red fox, Italy, 2011.
