# Supplementary material for: Group A Rotavirus Associated with Encephalitis in Red Fox
Source: Emerg Infect Dis. 2017 Sep;23(9):1535–8. doi: 10.3201/eid2309.170158 (PMC5572894; doi:10.3201/eid2309.170158)
Supplement: Technical Appendix — Additional methods used in study of group A rotavirus associated with encephalitis in red fox, Italy, 2011. [file 17-0158-Techapp-s1.pdf]

Article DOI: https://doi.org/10.3201/eid2309.170158

Group A Rotavirus Associated with Encephalitis in Red Fox

Technical Appendix

Additional Methods and Details

Viruses

During a national surveillance program for rabies in wildlife animals, a red fox (*Vulpes vulpes*) was captured in the Apennines of Piacenza province, Italy. The animal showed nervous disorders, including ataxia and head shaking. Due to bad general conditions, the fox was euthanized and the brain of the animal was collected and screened for a panel of neuro-pathogens, as requested by the Italian veterinarian health system.

Screening for neuro-pathogens

The animal was tested with a diagnostic panel specific for neuro-pathogens: rabies, canine distemper, Aujeszky's disease, leishmaniasis and flavivirus infections. Mouse inoculation test and Fluorescent Antibody Test (FAT) for rabies virus were performed according to the OIE guidelines (OIE 2013). Briefly, ten Specific Pathogen-Free (SPF) Swiss mice of 2 days of age ("suckling mice") and ten 17-day old mice ("weaning mice") were inoculated intra-cerebrally with 30 μL of clarified supernatant obtained from a 10% (w/v) homogenate of brain material made in an isotonic buffered solution containing antibiotics. Three mice were used as controls. The mice were observed daily, and each dead mouse was examined for rabies using the FAT assay. Detection of canine distemper virus, Aujeszky's disease virus, flavivirus and Leishmania infantum was performed by Reverse transcriptase-PCR (RT-PCR) or PCR as previously described (*1*–*4*).

Electron Microscopy (EM)

The brain homogenates of the naturally infected fox and the experimentally infected mice were submitted to negative staining electron microscopy using the Airfuge method (*5*). Viral particles were spun directly onto grids in an ultracentrifuge (82,000 x *g*, 15 min; Airfuge® with

fixed-angle rotor A-100/18 (Beckman Coulter, USA, California, Brea) and contrasted with phosphotungstic acid (PTA). The morphological identification of viral particles was obtained by observation with a FEI (Eindhoven, NL) Tecnai G2 Spirit Biotwin electron microscope at different magnifications (range, 20,500x to 43,000x).

Histology and immunohistochemistry (IHC)

A fragment of frozen cerebrum was fixed in 10% buffered formalin, paraffin-embedded and cut into serial sections of 3 μm thickness. A section was stained with hematoxylin-eosin (HE), whilst the other sections were incubated overnight with a polyclonal serum raised against RVA (IZSLER, Italy, Brescia), diluted 1:2000 and developed with Novolink™ polymer detection system (Leica, Germany, Wetzlar), using Vector® NovaRED™ substrate kit as chromogen.

Culture isolation, virus purification, RNA extraction, and electropherotype

Homogenate of the fox brain was used to infect confluent monolayers of Marc-145 cells (a highly homogenous MA-104 derived cell line) in Minimum Essential Medium (MEM), without and with 10 μg/mL trypsin.

Homogenates from the fox cerebral tissues, from the brain of mice infected with the fox homogenate and from Marc-145 infected cells, were purified by centrifugation at 35,000 x *g* for 2 h on a 24% sucrose layer at 4°C in an SW40 rotor (Beckman Coulter, USA, California, Brea). The pellets were suspended in 200 μL of MEM. Subsequently, the samples were treated with distilled water (1:1, v/v), for 15 min at room temperature to remove residual host cells. Non-encapsidated DNA was removed by digestion with 10 U of DNase I (Invitrogen, USA, California, Carlsbad), at 65°C for 10 minutes with 50 μl of 10X DNase I reaction buffer (Invitrogen).

Viral RNA was extracted using EuroGold RNA Pure solution (Euroclone, Italy, Milan) according to manufacturer’s protocol. Purified viral RNA was quantified spectrophotometrically (NanoQuant, Tecan) and stored at -20°C.

Electrophoresis of the extracted RNA was carried out in 7.5% polyacrylamide slab gels with a 3.5% stacking gel, using the discontinuous buffer system described by Laemmli (1970) (*6*) without sodium dodecyl sulfate. Electrophoresis was performed at 100 V for 16 to 18 h at

4°C. SilverXpress® Silver Staining Kit (Invitrogen), was applied to visualize RNA segments according to manufacturer's protocol.

Genome sequencing

Sequence-independent amplification of viral nucleic acids was performed, as previously described by Victoria JG et al. 2008 (*7*), by incubation of the extracted viral RNA with 5 pmol of primer K-8N (GAC CAT CTA GCG ACC TCC ACN NNN NNN N), and 0.5 mM of each deoxynucloside triphosphate (dNTP) at 75°C for 5 min. Subsequently, 5U of RNase inhibitor, 10 mM dithiothreitol, 1X first-strand buffer, and 200 U of SuperScript® II reverse transcriptase (Invitrogen) were added to the mixture and incubated at 25°C for 5 min, followed by an incubation at 37°C for 1 hr. A single round of double-strand (ds) DNA synthesis was then performed using Klenow fragment polymerase (New England Biolabs, USA, Massachusets, Ipswich): 20 µl of cDNA were heated at 95°C for 2 min and then cooled at 4°C in the presence of 0,5 pmol of primer K-8N and 1X NEB buffer, 0,125 U of DNA polymerase I, large (klenow) fragment. Reaction was performed at 37°C for 60 min. Finally, the reaction products were purified by NucleoSpin® gel and PCR clean-up (Macherey-Nagel, Germany, Düren) following manufacturer's procedure. PCR amplification of nucleic acids was then performed using primer K (GAC CAT CTA GCG ACC TCC AC), consisting of the fixed portion of the random primers K-8N. Five µl of the reaction described above was used in a total reaction volume of 25 µl containing 0,2 mM of each dNTP, 1X GoTaq® Reaction buffer, 0,8 pmol of primer K, and 0,1 U of GoTaq® DNA polymerase (Promega, USA, Wisconsin, Madison). Temperature cycling was performed as follows: 1 cycle at 95°C for 3 min, 25 cycles of denaturing at 95°C for 30 sec, annealing at 55°C for 30 sec, extension at 72°C for 1 min. An additional extension step at 72°C for 7 min was added to the end of the run. PCR was loaded on a 2% agarose gel electrophoresis where a smear of fragments was observed. Fragments larger than 500bp were purified by Nucleospin® gel and PCR clean-up (Macherey-Nagel) and used for subcloning into pCR™ 2.1 vector (Invitrogen) according to manufacturer's instruction.

Thirty-seven clones were sequenced using M13 primers. The sequences obtained from the clones covered around 14.6% of the genome (Figure 2 online Technical Appendix). The remaining genome sequences were obtained by amplification with specific primers. The primers were designed using the alignment of the sequences obtained from the clones and the genome sequence of the avian RVA strain PO-13 (online Technical Appendix Table 1). Reverse

transcription was performed using 1 μg of RNA with 10 pmol of random primers and 10 mM of each dNTP, heating at 65°C for 5 min followed by rapid cooling on ice. Then, 10 mM dithiothreitol and 1X first-strand buffer were added to the mixture and incubated at 25°C for 2 min. Subsequently, 200 U of SuperScript™ II reverse transcriptase (Invitrogen) was added to the 20-μL final mixture and then incubated at 25°C for 10 min and then at 42°C for 50 min and terminated at 70°C for 15 min. The PCR assays were performed in a Veriti Thermal Cycler (Applied Biosystems, USA, California, Foster City) using 1 μL of cDNA in a total reaction volume of 25 μL containing 0.2 mM of each dNTP, 1X GoTaq® reaction buffer, 1 pmol primer, and 0.1 U of GoTaq® DNA polymerase (Promega). Thermal cycling was performed as follows: 1 cycle at 95°C for 3 min, 35 cycles of denaturation at 95°C for 30 sec, annealing at 46-50°C for 30 sec and extension at 72°C for 1 min, and a final extension step at 72°C for 7 min. The PCR products were purified with the Nucleospin® gel and PCR clean-up Kit (Macherey-Nagel). Sequencing reactions were performed with fluorescent dideoxy chain-termination chemistry using the BigDye® Terminator v1.1 cycle sequencing kit (Applied Biosystems) on an automated sequencer (ABI PRISM® 3130 Automated Capillary DNA Sequencer, Applied Biosystems).

Finally, the 3'- and 5'-terminal sequences of the genome segments were determined by a primer ligation strategy (*8*), with minor modifications. Briefly, extracted RNA was ligated with a single amino-linked modified oligonucleotide (Linker: 5’-PO4-TTC CTT ATG CAG CTG ATC ACT CTG TGT CA-C6-NH2-3’) to the 3'-end of both strands of dsRNA. About 1 μg of viral RNA was added to 8 μL of 10X T4 RNA Ligase buffer (Promega), 40 μL of 40% PEG 8000, 2 μL OligoLinker (final concentration ≈ 1 μM), 20 U T4 RNA Ligase (Promega) in a final volume of 83 μL, incubated at room temperature for 2 hours and then stored at -20°C. Five μL of ligation product was used as template for a One-Step RT-PCR (Qiagen, Germany, Hilden) reaction with a first primer chosen inside the sequence of the virus (Online Technical Appendix Table 2) and the second primer complementary to the linker sequence (5’-TGA CAC AGA GTG ATC AGC-3'). Thermal cycling was performed as follows: reverse transcription at 45°C for 30 min, enzyme activation at 95°C for 15 min, gradual cooling down from 83°C to 65°C, 3 min for step (8 step), 45 cycles of denaturing at 94°C for 30 sec, annealing at 46-50°C for 30 sec and extension at 72°C for 1 min. A final extension step at 72°C for 10 min was added to the end of the run.

Sequence analysis

The sequences obtained were assembled and aligned using the SeqMan module of the DNASTAR software package (Lasergene, USA, Madison). Genotype assignment of each segment was performed using the RotaC v2.0 (http://rotac.regatools.be) automated genoptyping tool for RVAs. The assignment of the novel NSP4 genotype was approved by the Rotavirus Classification Working Group (RCWG). (https://rega.kuleuven.be/cev/viralmetagenomics/virus-classification/rcwg). The sequences of the genomic segments of RVA/Fox-wt/ITA/288356/2011 have been deposited in GenBank under accession numbers KT873803 through KT873813. Phylogenetic analysis was performed on both concatenated and separate nucleotide sequences obtained from the genomic segments using the Neighbor-Joining method p-distance model and bootstrap test of 1,000 replicates in MEGA 5 (http://www.megasoftware.net/).

References

1. Frisk AL, König M, Moritz A, Baumgärtner W. Detection of canine distemper virus nucleoprotein RNA by reverse transcription-PCR using serum, whole blood, and cerebrospinal fluid from dogs with distemper. J Clin Microbiol. 1999;37:3634–43.

2. Yoon HA, Eo SK, Aleyas AG, Park SO, Lee JH, Chae JS, et al. Molecular survey of latent pseudorabies virus infection in nervous tissues of slaughtered pigs by nested and real-time PCR. J Microbiol. 2005;43:430–6.

3. Scaramozzino N, Crance JM, Jouan A, DeBriel DA, Stoll F, Garin D. Comparison of flavivirus universal primer pairs and development of a rapid, highly sensitive heminested reverse transcription-PCR assay for detection of flaviviruses targeted to a conserved region of the NS5 gene sequences. J Clin Microbiol. 2001;39:1922–7. http://dx.doi.org/10.1128/JCM.39.5.1922-1927.2001

4. Galletti E, Bonilauri P, Bardasi L, Fontana MC, Ramini M, Renzi M, et al. Development of a minor groove binding probe based real-time PCR for the diagnosis and quantification of Leishmania infantum in dog specimens. Res Vet Sci. 2011;91:243–5. http://dx.doi.org/10.1016/j.rvsc.2011.01.004

5. Lavazza A, Pascucci S, Gelmetti D. Rod-shaped virus-like particles in intestinal contents of three avian species. Vet Rec. 1990;126:581.

6. Laemmli UK. Cleavage of structural proteins during the assembly of the head of bacteriophage T4. Nature. 1970;227:680–5. http://dx.doi.org/10.1038/227680a0

7. Victoria JG, Kapoor A, Dupuis K, Schnurr DP, Delwart EL. Rapid identification of known and new RNA viruses from animal tissues. PLoS Pathog. 2008;4:e1000163. http://dx.doi.org/10.1371/journal.ppat.1000163

8. Matthijnssens J, Rahman M, Van Ranst M. Two out of the 11 genes of an unusual human G6P[6] rotavirus isolate are of bovine origin. J Gen Virol. 2008;89:2630–5. http://dx.doi.org/10.1099/vir.0.2008/003780-0

9. Zhang M, Zeng CQ, Dong Y, Ball JM, Saif LJ, Morris AP, et al. Mutations in rotavirus nonstructural glycoprotein NSP4 are associated with altered virus virulence. J Virol. 1998;72:3666–72.

10. Mohan KV, Dermody TS, Atreya CD. Mutations selected in rotavirus enterotoxin NSP4 depend on the context of its expression. Virology. 2000;275:125–32. http://dx.doi.org/10.1006/viro.2000.0484

11. Jagannath MR, Kesavulu MM, Deepa R, Sastri PN, Kumar SS, Suguna K, et al. N- and C-terminal cooperation in rotavirus enterotoxin: novel mechanism of modulation of the properties of a multifunctional protein by a structurally and functionally overlapping conformational domain. J Virol. 2006;80:412–25. http://dx.doi.org/10.1128/JVI.80.1.412-425.2006

Technical Appendix Table 1. Primers used for amplification and sequencing of the genome segments of the strain RVA/Fox-wt/ITA/288356/2011

Segment number (encoded protein)	Name	Sequence (5' → 3')	position (nt)	Amplicon size
1 (VP1)	VP1_9F	GGCTATTAAAGCTATACGATG	1 - 21	740
	VP1_18R	AGTTGATCTGTTCGAATATG	740 - 721	
	VP1_11F	TAACTTGGGCAAATTCATCA	629 - 648	330
	VP1_12R	GGTGCATCTATCCAGTTCCTT	959 - 939	
	VP1_2F	CACAACGGAAGAACTTGAAC	854 - 817	385
	VP1_3R	TTGTCTGGACTCACCATTCG	1239 - 1220	
	VP1_13F	GTTGATTCAGAACTAGCTGG	1180 - 1199	516
	VP1_14R	CGAGTGTTTCTTTAACTTTCCT	1696 - 1675	
	VP1_15F	ATACTGACGTATCGCAATGG	1574 - 1593	804
	VP1_16R	CGCTGTAAATACACATCGTC	2378 - 2359	
	VP1_8F	GTACTGACTACGAACTCAACG	2284 - 2304	1016
	VP1_10F	TCATATCTTAGCGCCTTAATC	3300 - 3280	
2 (VP2)	VP2_20F	AGAAGGAGGTGCAATATGAA	316 - 335	275
	VP2_21R	CGAGTACTTGCGAATAAAGG	591 - 572	
	VP2_2F	TGGATGGTATTGGAAATTG	500 - 518	695
	VP2_5R	TTCGCTGCTTGAGAGTTAAT	1195 - 1176	
	VP2-10F	TCGAGTCAATCTGGGATGCT	1009 - 1028	491
	VP2-11R	TGTGGTTGACAAAATGTAGC	1500 - 1481	
	VP2-16F	CAAAACGATCATAGCGTGTA	1205 - 1224	765
	VP2_7R	ATACAGGTCCAGATGGTTTG	1970 - 1951	
	VP2_12F	AGAGCCACGTACACTGTTTC	1853 - 1872	885
	VP2_9R	GGTCATATCACCATTTACG	2738 - 2720	
3 (VP3)	VP3_5F	GGCTATTAAAGCTACATGAG	1 - 20	659
	VP3_1R	TAGTGTCTTTTGGCACCTTC	659 - 640	
	VP3_2F	TCAGATAGAAATGGCGTTG	1067 - 1085	944
	VP3_3R	TGCATATAGCAATTCTGGTG	2011 - 1992	
4 (VP4)	VP4_5F	GGCTATAAAATGGCTTCTC	1 - 19	769
	VP4_4R	TTGAAATCACTATGTCTTCG	769 - 750	
	VP4_11F	ATGCCAGTTGAACGATTAGT	703 - 722	470
	VP4_12R	TCCAAGTGGAAGTTGAAAAT	1173 - 1154	
	VP4_8F	AGGCATTCAAAAACATGGTA	1076 - 1095	520
	VP4_1R	TCCAGAAAACATTGAGAAC	1596 - 1578	
	VP4_13F	GGAAATAGCAATGTCTCAGC	1527 - 1546	389
	VP4_14R	TTTAATACAGCAGCCGAAAT	1916 - 1896	
	VP4_2F	AGAGTGAAAGAATTCGCAAC	1846 - 1865	503
	VP4_6R	GGTCACATCCTCATAGAC	2349 - 2332	
5 (NSP1)	NSP1_5R	GGCTTTTAAAAGCTGAACAG	1 - 20	661
	NSP1_1R	CTCTGAGGCTCATAATGTCCA	661 - 641	
	NSP1_9F	ATTGTTTCCTGCCAACAAAT	550 - 569	606
	NSP1_10R	ATGTCTTCCAAATTGTCCAG	1156 - 1137	
	NSP1_4F	GGATGGACTCACTTCTCATAC	1092 - 1112	778
	NSP1_6R	GGTCTCATAAAGCCATTTTG	1870 - 1851	
6 (VP6)	VP6_7F	CTGATTCGCTAAGACAGCTT	361 - 381	690
	VP6_8R	AATATGGCTCATTTGCATCT	1051 - 1031	
7 (NSP3)	NSP3_5R	GGCTTTTAAAGTCACATCGAG	1 - 21	526
	NSP3_7R	ATCACTTCCCCACGTTTGAG	526 - 506	
	NSP3_9F	AGGATGATGCTTTCTGCTAA	381 - 400	711
	NSP3_6R	GGTCACATAAAGTCTCGTGC	1092 - 1073	
8 (VP7)	VP7_3F	GGCATTAAAATCAGTAATTTTC	1 - 22	189
	VP7_1R	GCGGCAAAAACAATAGTAAG	189 - 170	
	VP7_2F	GACACGCATTAATTGGAAG	910 - 928	155
	VP7_4R	GGTCACATCAATCCTTCAC	1065 - 1047	
9 (NSP2)	NSP2_3R	GGCTTTTAAAGCGTCTCGG	1 - 19	889
	NSP2_1R	CTAGTTTCATCCCATTCAGC	889 - 870	
	NSP2_2F	ACCATTTAAAGGAGTCACG	935 - 954	107
	NSP2_4R	GGTCACATAAAGCGCTTTCAATTC	1042 - 1019	
10 (NSP4)	NSP4_3F	GGAAAGATGGAGAACGCTAC	35 - 54	257
	NSP4_6R	GAGACCACTTCAGTTTTTGAG	292 - 312	
	NSP4_7F	GGATTTAAAGTAGTGGGGGT	230 - 249	444

Segment number (encoded protein)	Name	Sequence (5' → 3')	position (nt)	Amplicon size
	NSP4_4R	GGTACCAGGGATTAAGTCTTC	674 - 654	
11 (NSP5)	NSP5_2F	GGCTTTTAAAGCGCTACAGTG	1 - 21	311
	NSP5_5R	TCTGCTGAGCTAGCATCCAA	311 - 292	

Technical Appendix Table 2. Primers used for the determination of the 5' and 3' terminal sequences

Segment number (encoded protein)	Name	Sequence (5'-3')	position (nt)
1 (VP1)	VP1_19R	CAACTGTGGCATATTCCTTT	304 - 285
	VP1_20F	TAATGTCACTCGGTGTACCA	2906 - 2925
2 (VP2)	VP2_19R	GTCTCGTTAGGTTGAAATGA	376 - 358
	VP2_15F	ACTGGACGCTACAGTCTTTG	2399 - 2418
3 (VP3)	VP3_7F	CAGGCAAAATGTATTCTTCA	410 - 391
	VP3_8F	GCATTAAATTTATTAACTCAATACACA	2276 - 1302
4 (VP4)	VP4_9R	AGCATATTTCTTATTCGTTGC	318 - 298
	VP4_10F	CCAACGCGTCAGTATAGAAT	1996 - 2015
5 (NSP1)	NSP1_11R	CACAAGCAAAAACATTATGG	261 - 242
	NSP1_12F	CACAAGAAATGAGAAGAAAAA	1576 - 1596
6 (VP6)	VP6_9R	CTCACGCACTATCTCATCAA	333 - 614
	VP6_10F	CAGTTCTGTCAGATGCAAAT	1022 - 1041
7 (NSP3)	NSP3_11R	GATTCACTGACGCCATTCT	315 - 297
	NSP3_12F	GGAATGGAAATTCAACTCAA	791 - 810
8 (VP7)	VP7_5R	AATGCATAGACTAGAGACTAAACC	317 - 284
	VP7_6F	CAATTATTCAGTCGCATCG	769 - 787
9 (NSP2)	NSP2_6R	AAAGTTCATCCCTCTGGAAT	258 - 239
	NSP2_7F	CAATCCAAAAGAGAAATCCA	782 - 801
10 (NSP4)	NSP4_8R	GAGCCAAATGTCTTCTTATACA	293 - 271
	NSP4_9F	ACAAAACGGGAACTAGAACA	388 - 407
11 (NSP5)	NSP5_5R	TCTGCTGAGCTAGCATCCAA	311 - 292
	NSP5_6F	GGCTCATCAGCTAAATTAACA	441 - 462

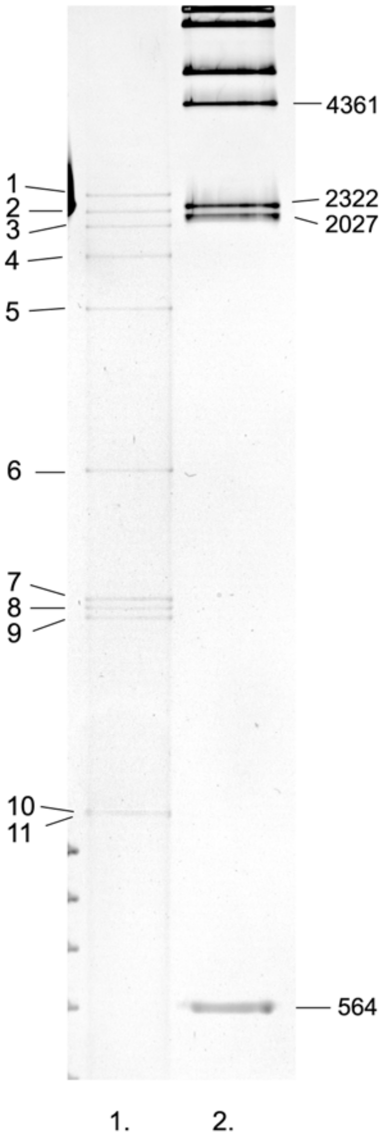

Technical Appendix Figure 1. Electrophoretic migration pattern of genomic RNA segments from the strain Fox-288356 amplified on culture cells on 7,5% polyacrylamide gel (lane 1). The segmented viral genome was characterized by a 5-1-3-2 profile with co-migration of segment 10 and 11. Gene segments are numbered on the left. The molecular marker was load on lane 2.

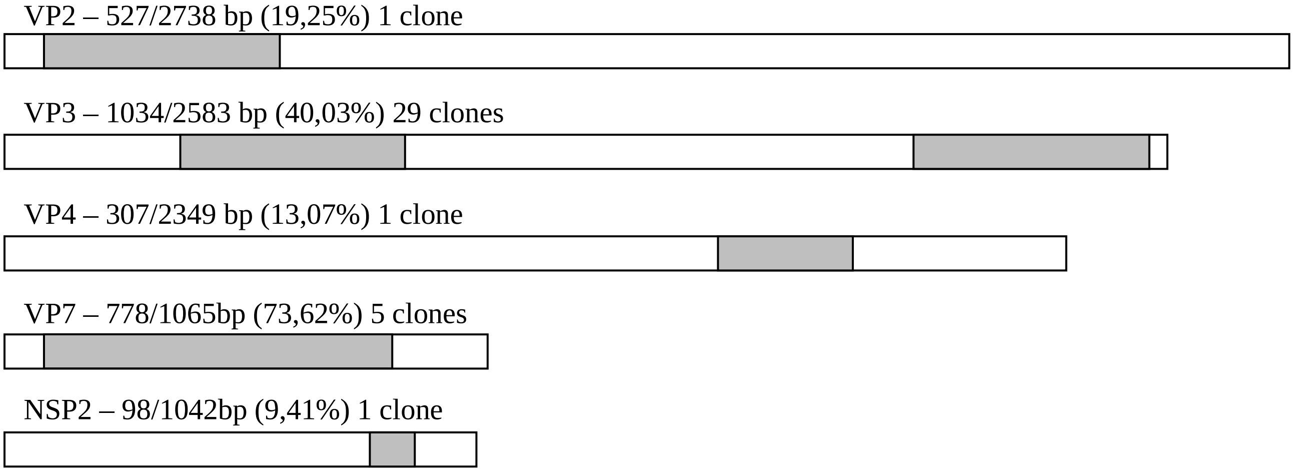

Technical Appendix Figure 2. Schematic representation of the clones obtained by the SISPA method.

Technical Appendix Figure 3 (following pages). Phylogenetic analysis of the genomic segments of the Fox-288356 strain. VP1 (A), VP2 (B), VP3 (C), VP4 (D), VP6 (E), VP7 (F), NSP1 (G), NSP2 (H), NSP3 (I), NSP4 (J), NSP5 (K). All the genomic segments are correlated to the strain RVA-PO-13 and clustered with the avian RVA. Bootstrap values >70% (1,000 replicates) are indicated. Reference sequences are identified by strain name and GenBank accession no.

(A) VP1

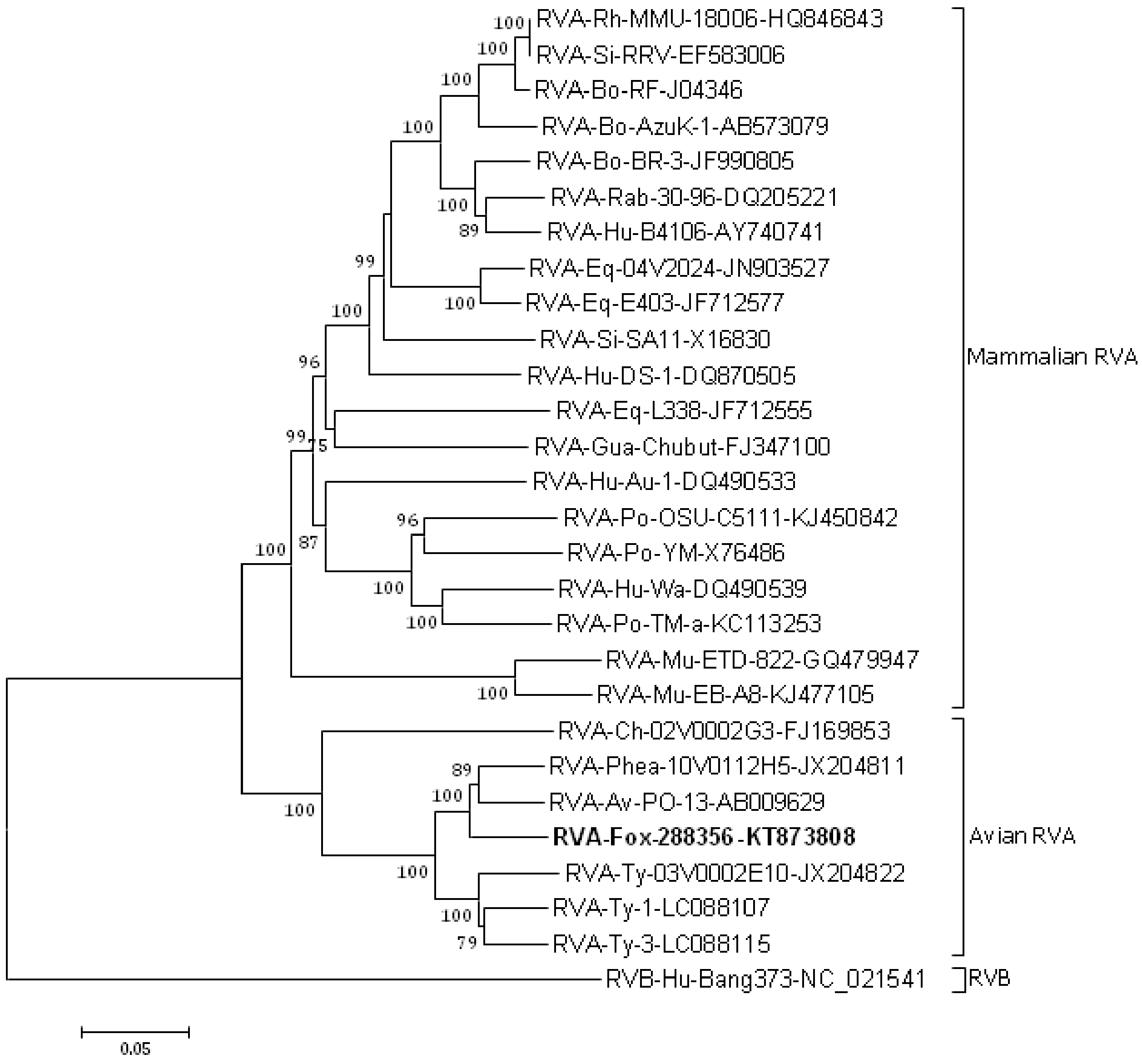

(B) VP2

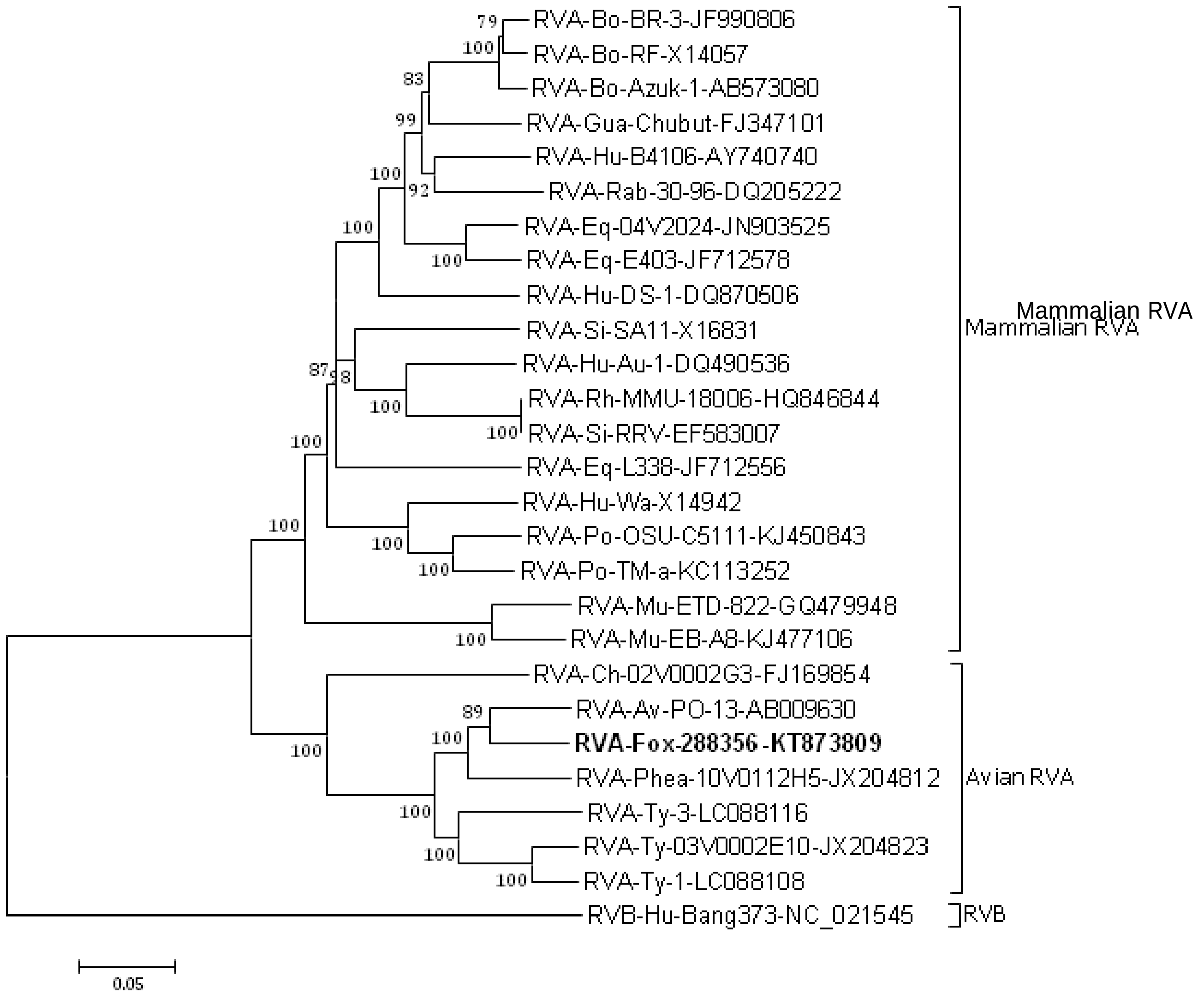

RVA-Bo-BR-3-JF990806
RVA-Bo-RF-X14057
RVA-Bo-Azuk-1-AB573080
RVA-Gua-Chubut-FJ347101
RVA-Hu-B4106-AY740740
RVA-Rab-30-96-DQ205222
RVA-Eq-04V2024-JN903525
RVA-Eq-E403-JF712578
RVA-Hu-DS-1-DQ870506
RVA-Si-SA11-X16831
RVA-Hu-Au-1-DQ490536
RVA-Rh-MMU-18006-HQ846844
RVA-Si-RRV-EF583007
RVA-Eq-L338-JF712556
RVA-Hu-Wa-X14942
RVA-Po-OSU-C5111-KJ450843
RVA-Po-TM-a-KC113252
RVA-Mu-ETD-822-GQ479948
RVA-Mu-EB-A8-KJ477106
RVA-Ch-02V0002G3-FJ169854
RVA-Av-PO-13-AB009630
RVA-Fox-288356-KT873809
RVA-Phea-10V0112H5-JX204812
RVA-Ty-3-LC088116
RVA-Ty-03V0002E10-JX204823
RVA-Ty-1-LC088108
RVB-Hu-Bang373-NC_021545
Mammalian RVA
Mammalian RVA
Avian RVA
RVB
0.05

(C) VP3

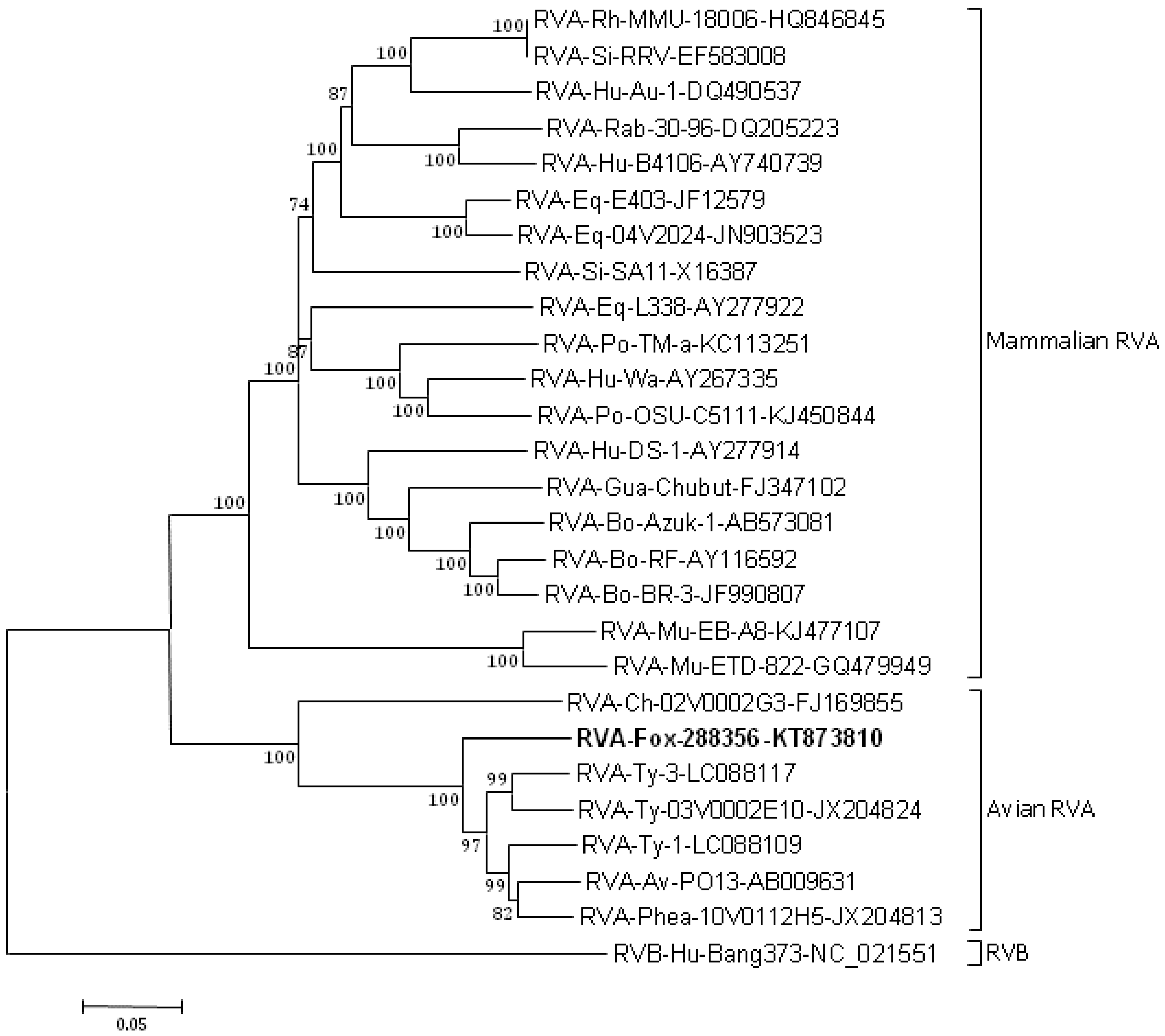
RVA-Rh-MMU-18006-HQ846845
RVA-Si-RRV-EF583008
RVA-Hu-Au-1-DQ490537
RVA-Rab-30-96-DQ205223
RVA-Hu-B4106-AY740739
RVA-Eq-E403-JF12579
RVA-Eq-04V2024-JN903523
RVA-Si-SA11-X16387
RVA-Eq-L338-AY277922
RVA-Po-TM-a-KC113251
RVA-Hu-Wa-AY267335
RVA-Po-OSU-C5111-KJ450844
RVA-Hu-DS-1-AY277914
RVA-Gua-Chubut-FJ347102
RVA-Bo-Azuk-1-AB573081
RVA-Bo-RF-AY116592
RVA-Bo-BR-3-JF990807
RVA-Mu-EB-A8-KJ477107
RVA-Mu-ETD-822-GQ479949
RVA-Ch-02V0002G3-FJ169855
RVA-Fox-288356-KT873810
RVA-Ty-3-LC088117
RVA-Ty-03V0002E10-JX204824
RVA-Ty-1-LC088109
RVA-Av-PO13-AB009631
RVA-Phea-10V0112H5-JX204813
RVB-Hu-Bang373-NC_021551
Mammalian RVA
Avian RVA
RVB
0.05

(D) VP4

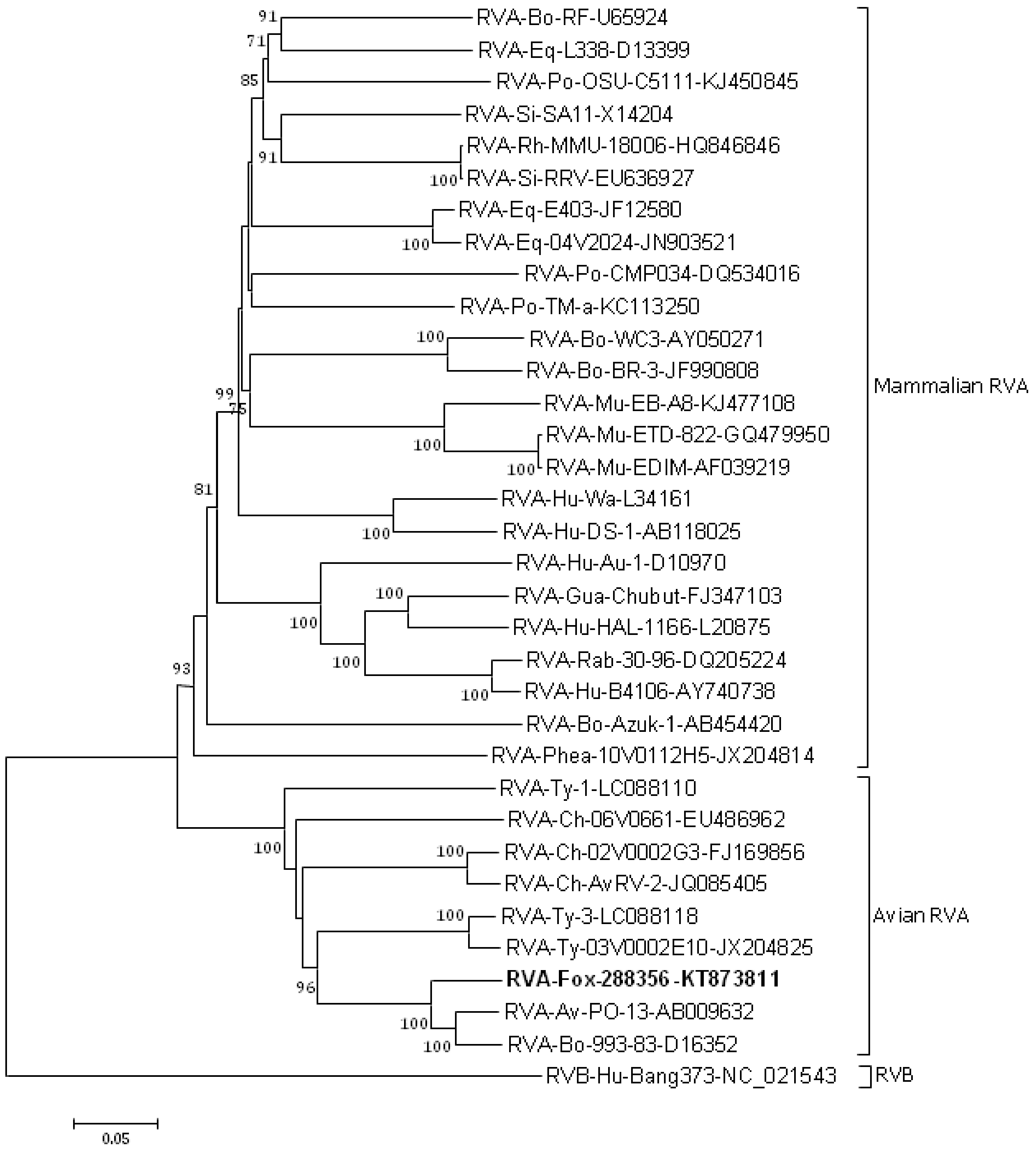

RVA-Bo-RF-U65924
RVA-Eq-L338-D13399
RVA-Po-OSU-C5111-KJ450845
RVA-Si-SA11-X14204
RVA-Rh-MMU-18006-HQ846846
RVA-Si-RRV-EU636927
RVA-Eq-E403-JF12580
RVA-Eq-04V2024-JN903521
RVA-Po-CMP034-DQ534016
RVA-Po-TM-a-KC113250
RVA-Bo-WC3-AY050271
RVA-Bo-BR-3-JF990808
RVA-Mu-EB-A8-KJ477108
RVA-Mu-ETD-822-GQ479950
RVA-Mu-EDIM-AF039219
RVA-Hu-Wa-L34161
RVA-Hu-DS-1-AB118025
RVA-Hu-Au-1-D10970
RVA-Gua-Chubut-FJ347103
RVA-Hu-HAL-1166-L20875
RVA-Rab-30-96-DQ205224
RVA-Hu-B4106-AY740738
RVA-Bo-Azuk-1-AB454420
RVA-Phea-10V0112H5-JX204814
RVA-Ty-1-LC088110
RVA-Ch-06V0661-EU486962
RVA-Ch-02V0002G3-FJ169856
RVA-Ch-AvRV-2-JQ085405
RVA-Ty-3-LC088118
RVA-Ty-03V0002E10-JX204825
RVA-Fox-288356-KT873811
RVA-Av-PO-13-AB009632
RVA-Bo-993-83-D16352
RVB-Hu-Bang373-NC_021543
Mammalian RVA
Avian RVA
RVB
0.05

(E) VP6

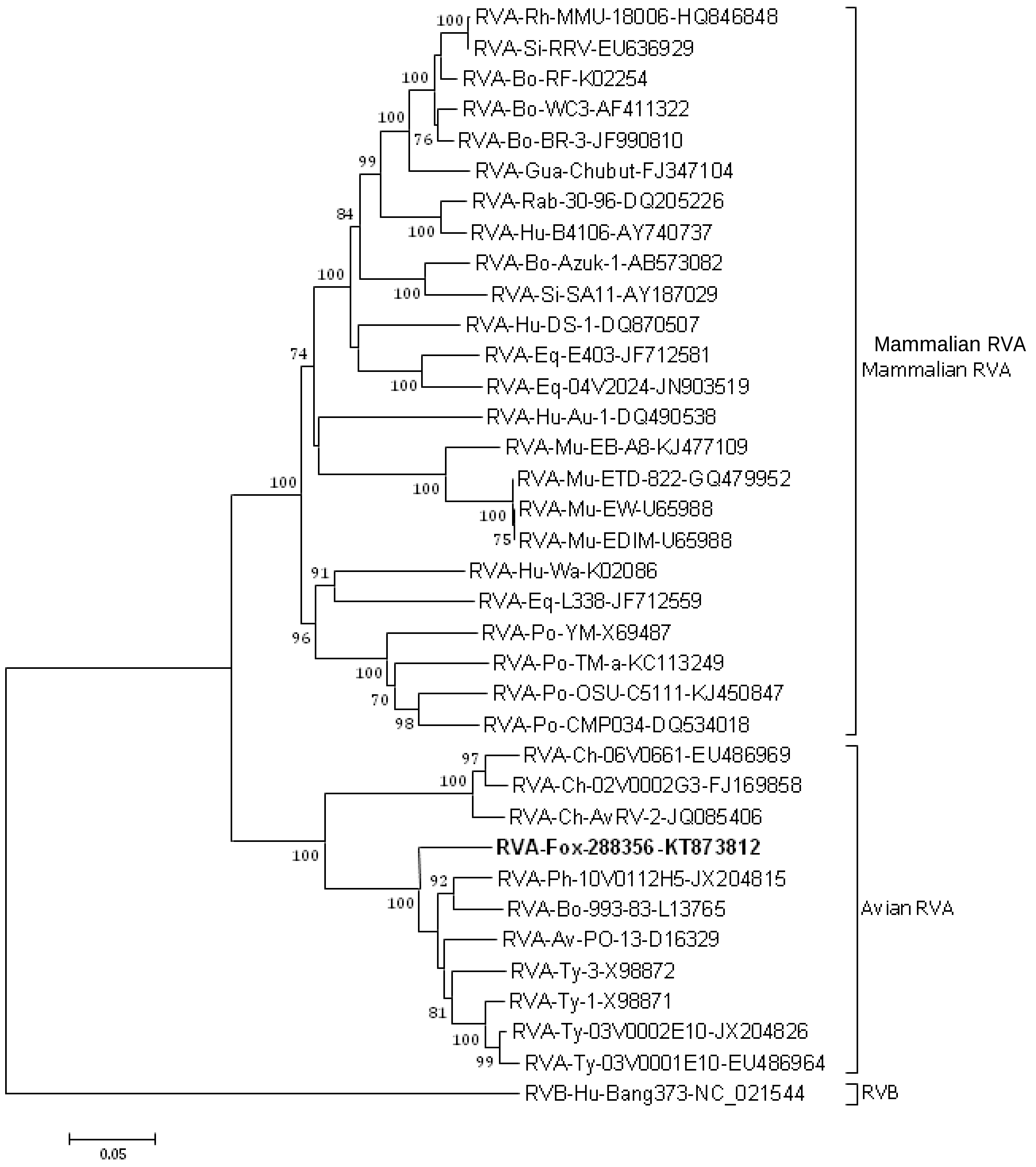

RVA-Rh-MMU-18006-HQ846848
RVA-Si-RRV-EU636929
RVA-Bo-RF-K02254
RVA-Bo-WC3-AF411322
RVA-Bo-BR-3-JF990810
RVA-Gua-Chubut-FJ347104
RVA-Rab-30-96-DQ205226
RVA-Hu-B4106-AY740737
RVA-Bo-Azuk-1-AB573082
RVA-Si-SA11-AY187029
RVA-Hu-DS-1-DQ870507
RVA-Eq-E403-JF712581
RVA-Eq-04V2024-JN903519
RVA-Hu-Au-1-DQ490538
RVA-Mu-EB-A8-KJ477109
RVA-Mu-ETD-822-GQ479952
RVA-Mu-EW-U65988
RVA-Mu-EDIM-U65988
RVA-Hu-Wa-K02086
RVA-Eq-L338-JF712559
RVA-Po-YM-X69487
RVA-Po-TM-a-KC113249
RVA-Po-OSU-C5111-KJ450847
RVA-Po-CMP034-DQ534018
RVA-Ch-06V0661-EU486969
RVA-Ch-02V0002G3-FJ169858
RVA-Ch-AvRV-2-JQ085406
RVA-Fox-288356-KT873812
RVA-Ph-10V0112H5-JX204815
RVA-Bo-993-83-L13765
RVA-Av-PO-13-D16329
RVA-Ty-3-X98872
RVA-Ty-1-X98871
RVA-Ty-03V0002E10-JX204826
RVA-Ty-03V0001E10-EU486964
RVB-Hu-Bang373-NC_021544
Mammalian RVA
Mammalian RVA
Avian RVA
RVB
0.05

(F) VP7

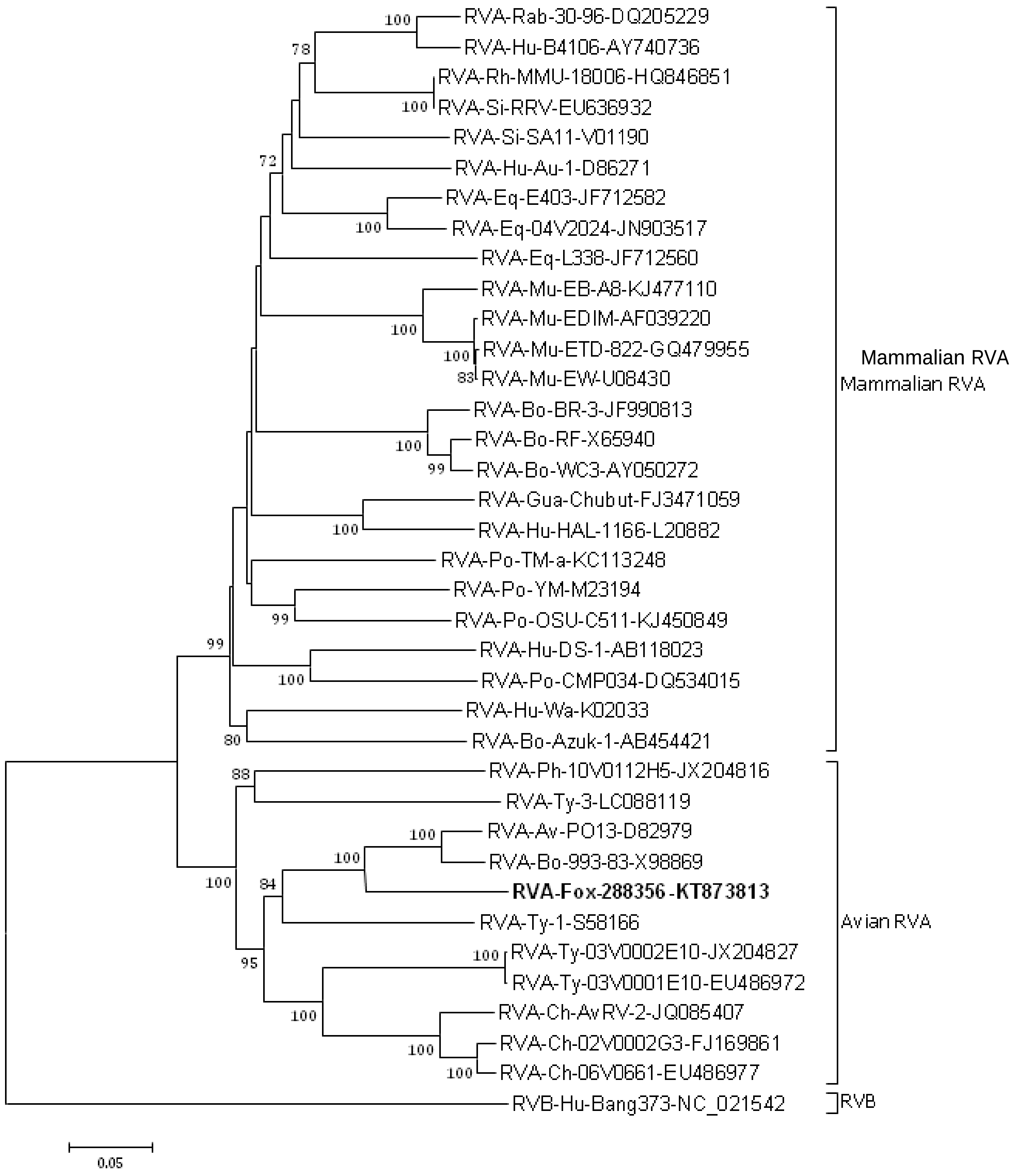

100
RVA-Rab-30-96-DQ205229
78
RVA-Hu-B4106-AY740736
RVA-Rh-MMU-18006-HQ846851
100
RVA-Si-RRV-EU636932
RVA-Si-SA11-V01190
72
RVA-Hu-Au-1-D86271
RVA-Eq-E403-JF712582
100
RVA-Eq-04V2024-JN903517
RVA-Eq-L338-JF712560
RVA-Mu-EB-A8-KJ477110
100
RVA-Mu-EDIM-AF039220
100
RVA-Mu-ETD-822-GQ479955
83
RVA-Mu-EW-U08430
RVA-Bo-BR-3-JF990813
100
RVA-Bo-RF-X65940
99
RVA-Bo-WC3-AY050272
RVA-Gua-Chubut-FJ3471059
100
RVA-Hu-HAL-1166-L20882
RVA-Po-TM-a-KC113248
RVA-Po-YM-M23194
99
RVA-Po-OSU-C511-KJ450849
99
RVA-Hu-DS-1-AB118023
100
RVA-Po-CMP034-DQ534015
RVA-Hu-Wa-K02033
80
RVA-Bo-Azuk-1-AB454421
88
RVA-Ph-10V0112H5-JX204816
RVA-Ty-3-LC088119
100
RVA-Av-PO13-D82979
100
RVA-Bo-993-83-X98869
100
84
RVA-Fox-288356-KT873813
RVA-Ty-1-S58166
100
RVA-Ty-03V0002E10-JX204827
95
RVA-Ty-03V0001E10-EU486972
100
RVA-Ch-AvRV-2-JQ085407
100
RVA-Ch-02V0002G3-FJ169861
100
RVA-Ch-06V0661-EU486977
RVB-Hu-Bang373-NC_021542
0.05
Mammalian RVA
Mammalian RVA
Avian RVA
RVB

(G) NSP1

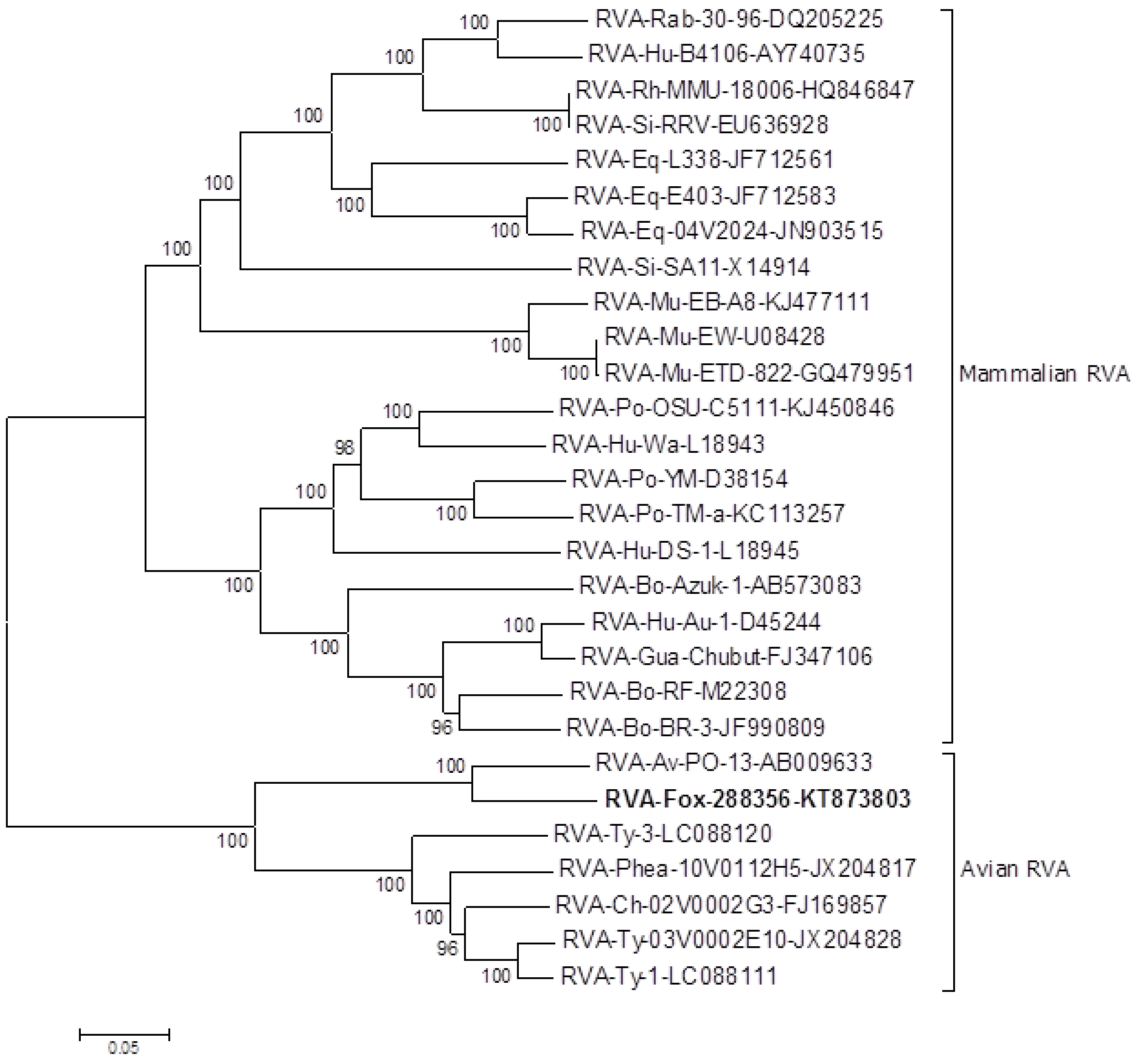
RVA-Rab-30-96-DQ205225
RVA-Hu-B4106-AY740735
RVA-Rh-MMU-18006-HQ846847
RVA-Si-RRV-EU636928
RVA-Eq-L338-JF712561
RVA-Eq-E403-JF712583
RVA-Eq-04V2024-JN903515
RVA-Si-SA11-X14914
RVA-Mu-EB-A8-KJ477111
RVA-Mu-EW-U08428
RVA-Mu-ETD-822-GQ479951
RVA-Po-OSU-C5111-KJ450846
RVA-Hu-Wa-L18943
RVA-Po-YM-D38154
RVA-Po-TM-a-KC113257
RVA-Hu-DS-1-L18945
RVA-Bo-Azuk-1-AB573083
RVA-Hu-Au-1-D45244
RVA-Gua-Chubut-FJ347106
RVA-Bo-RF-M22308
RVA-Bo-BR-3-JF990809
RVA-Av-PO-13-AB009633
RVA-Fox-288356-KT873803
RVA-Ty-3-LC088120
RVA-Phea-10V0112H5-JX204817
RVA-Ch-02V0002G3-FJ169857
RVA-Ty-03V0002E10-JX204828
RVA-Ty-1-LC088111
Mammalian RVA
Avian RVA
0.05

(H) NSP2

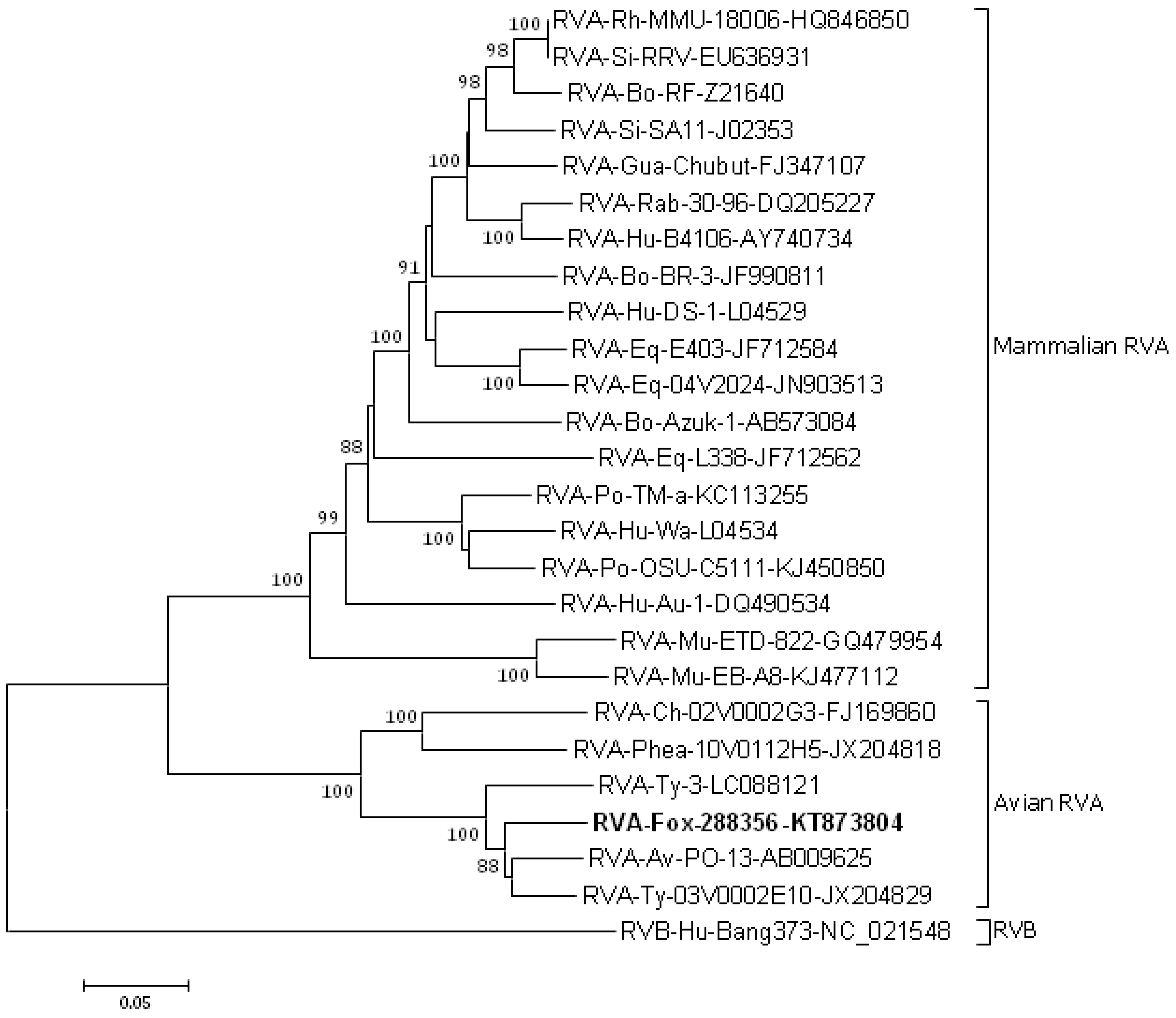
RVA-Rh-MMU-18006-HQ846850
RVA-Si-RRV-EU636931
RVA-Bo-RF-Z21640
RVA-Si-SA11-J02353
RVA-Gua-Chubut-FJ347107
RVA-Rab-30-96-DQ205227
RVA-Hu-B4106-AY740734
RVA-Bo-BR-3-JF990811
RVA-Hu-DS-1-L04529
RVA-Eq-E403-JF712584
RVA-Eq-04V2024-JN903513
RVA-Bo-Azuk-1-AB573084
RVA-Eq-L338-JF712562
RVA-Po-TM-a-KC113255
RVA-Hu-Wa-L04534
RVA-Po-OSU-C5111-KJ450850
RVA-Hu-Au-1-DQ490534
RVA-Mu-ETD-822-GQ479954
RVA-Mu-EB-A8-KJ477112
RVA-Ch-02V0002G3-FJ169860
RVA-Phea-10V0112H5-JX204818
RVA-Ty-3-LC088121
RVA-Fox-288356-KT873804
RVA-Av-PO-13-AB009625
RVA-Ty-03V0002E10-JX204829
RVB-Hu-Bang373-NC_021548
Mammalian RVA
Avian RVA
RVB
0.05

(I) NSP3

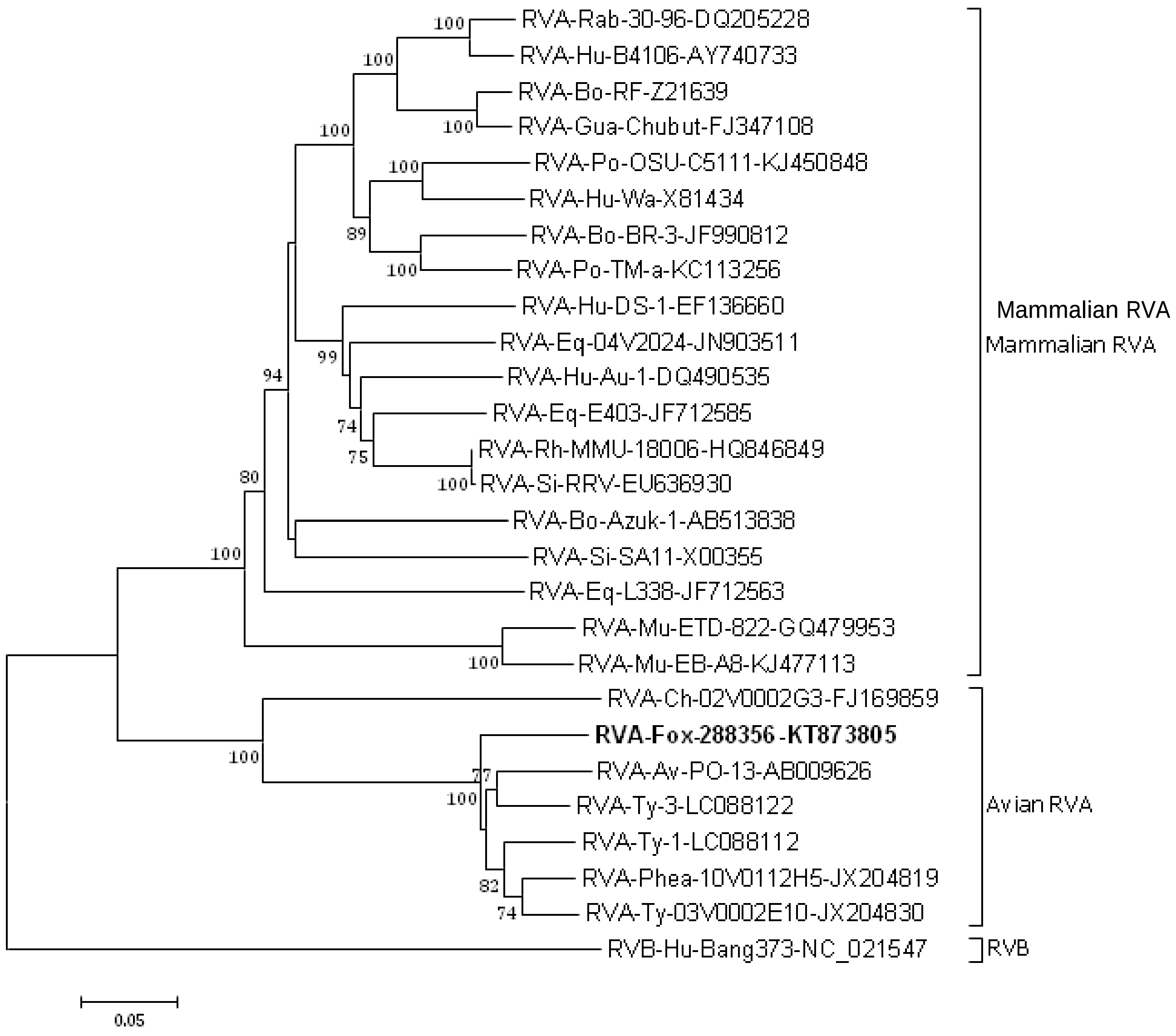
RVA-Rab-30-96-DQ205228
RVA-Hu-B4106-AY740733
RVA-Bo-RF-Z21639
RVA-Gua-Chubut-FJ347108
RVA-Po-OSU-C5111-KJ450848
RVA-Hu-Wa-X81434
RVA-Bo-BR-3-JF990812
RVA-Po-TM-a-KC113256
RVA-Hu-DS-1-EF136660
RVA-Eq-04V2024-JN903511
RVA-Hu-Au-1-DQ490535
RVA-Eq-E403-JF712585
RVA-Rh-MMU-18006-HQ846849
RVA-Si-RRV-EU636930
RVA-Bo-Azuk-1-AB513838
RVA-Si-SA11-X00355
RVA-Eq-L338-JF712563
RVA-Mu-ETD-822-GQ479953
RVA-Mu-EB-A8-KJ477113
RVA-Ch-02V0002G3-FJ169859
RVA-Fox-288356-KT873805
RVA-Av-PO-13-AB009626
RVA-Ty-3-LC088122
RVA-Ty-1-LC088112
RVA-Phea-10V0112H5-JX204819
RVA-Ty-03V0002E10-JX204830
RVB-Hu-Bang373-NC_021547
Mammalian RVA
Mammalian RVA
Avian RVA
RVB
0.05

(J) NSP4

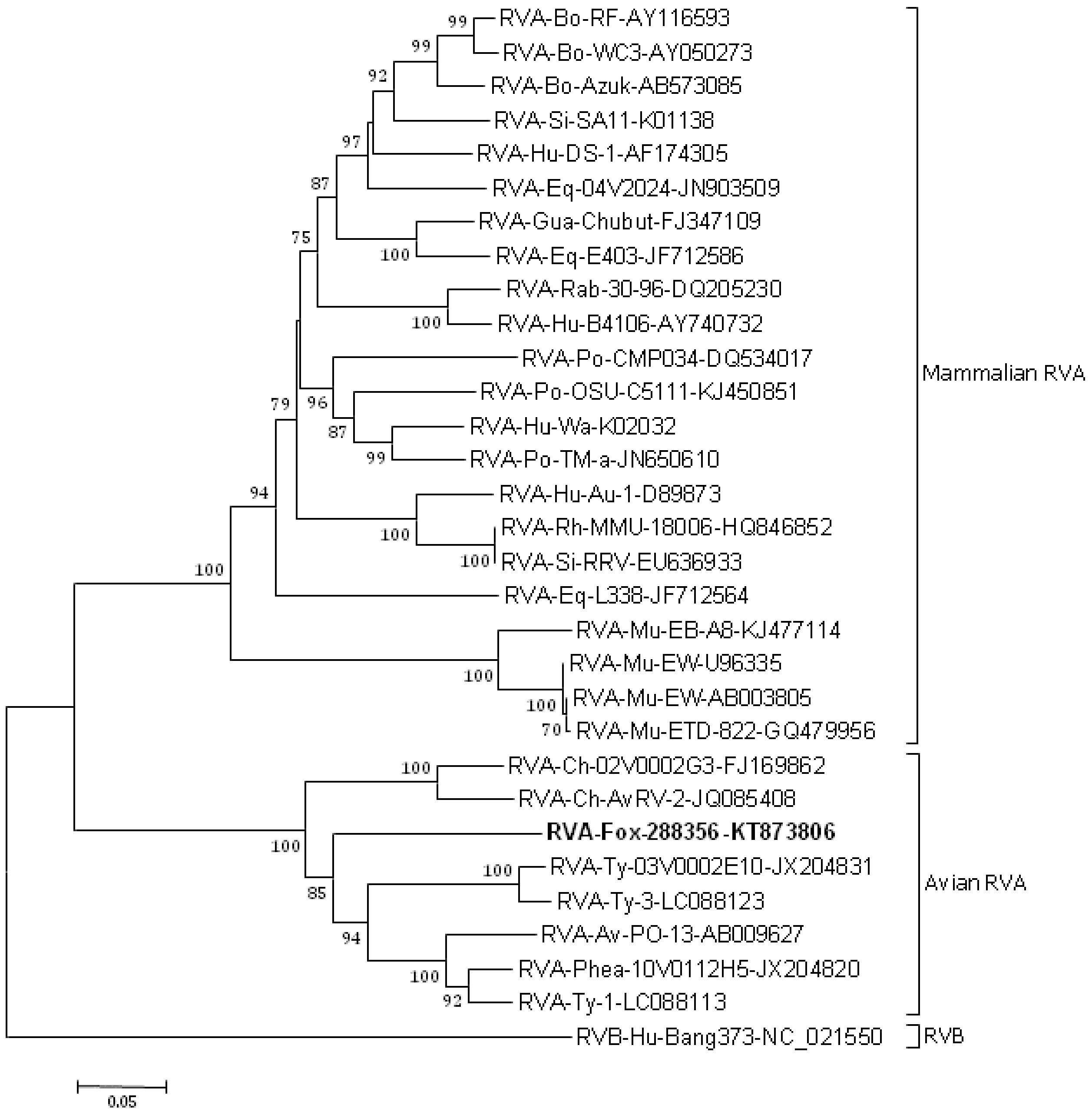
RVA-Bo-RF-AY116593
RVA-Bo-WC3-AY050273
RVA-Bo-Azuk-AB573085
RVA-Si-SA11-K01138
RVA-Hu-DS-1-AF174305
RVA-Eq-04V2024-JN903509
RVA-Gua-Chubut-FJ347109
RVA-Eq-E403-JF712586
RVA-Rab-30-96-DQ205230
RVA-Hu-B4106-AY740732
RVA-Po-CMP034-DQ534017
RVA-Po-OSU-C5111-KJ450851
RVA-Hu-Wa-K02032
RVA-Po-TM-a-JN650610
RVA-Hu-Au-1-D89873
RVA-Rh-MMU-18006-HQ846852
RVA-Si-RRV-EU636933
RVA-Eq-L338-JF712564
RVA-Mu-EB-A8-KJ477114
RVA-Mu-EW-U96335
RVA-Mu-EW-AB003805
RVA-Mu-ETD-822-GQ479956
RVA-Ch-02V0002G3-FJ169862
RVA-Ch-AvRV-2-JQ085408
RVA-Fox-288356-KT873806
RVA-Ty-03V0002E10-JX204831
RVA-Ty-3-LC088123
RVA-Av-PO-13-AB009627
RVA-Phea-10V0112H5-JX204820
RVA-Ty-1-LC088113
RVB-Hu-Bang373-NC_021550
Mammalian RVA
Avian RVA
RVB
0.05

(K) NSP5

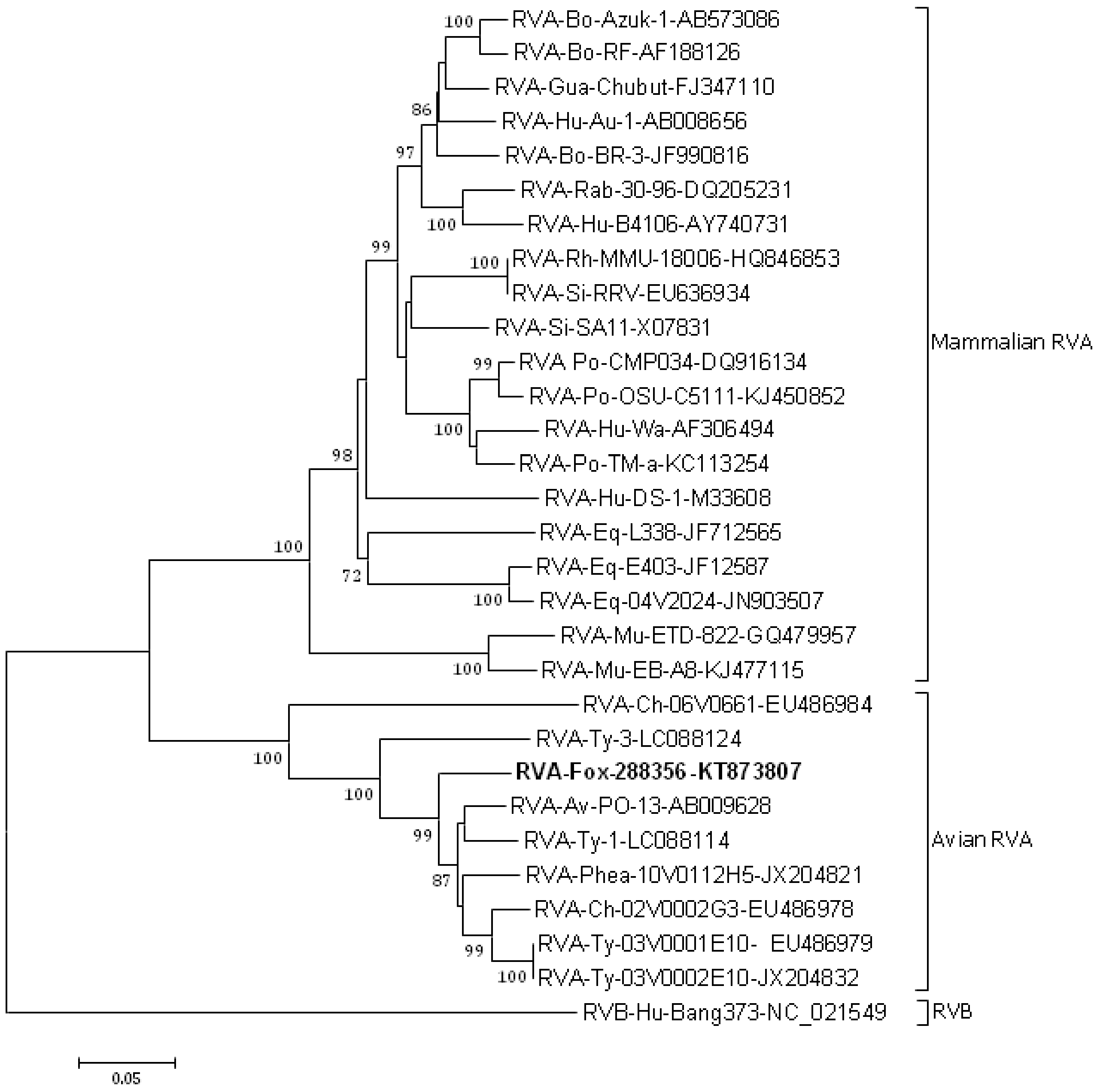
RVA-Bo-Azuk-1-AB573086
RVA-Bo-RF-AF188126
RVA-Gua-Chubut-FJ347110
RVA-Hu-Au-1-AB008656
RVA-Bo-BR-3-JF990816
RVA-Rab-30-96-DQ205231
RVA-Hu-B4106-AY740731
RVA-Rh-MMU-18006-HQ846853
RVA-Si-RRV-EU636934
RVA-Si-SA11-X07831
RVA Po-CMP034-DQ916134
RVA-Po-OSU-C5111-KJ450852
RVA-Hu-Wa-AF306494
RVA-Po-TM-a-KC113254
RVA-Hu-DS-1-M33608
RVA-Eq-L338-JF712565
RVA-Eq-E403-JF12587
RVA-Eq-04V2024-JN903507
RVA-Mu-ETD-822-GQ479957
RVA-Mu-EB-A8-KJ477115
RVA-Ch-06V0661-EU486984
RVA-Ty-3-LC088124
RVA-Fox-288356-KT873807
RVA-Av-PO-13-AB009628
RVA-Ty-1-LC088114
RVA-Phea-10V0112H5-JX204821
RVA-Ch-02V0002G3-EU486978
RVA-Ty-03V0001E10- EU486979
RVA-Ty-03V0002E10-JX204832
RVB-Hu-Bang373-NC_021549
Mammalian RVA
Avian RVA
RVB
100
86
97
100
99
100
99
100
98
100
72
100
100
100
100
99
87
99
100
0.05

```
                  10         20         30         40         50
VP7 288356   MYGTECTILL IEIIFYFFTA VVVYDVIHKM ANSPTLCIIV LTIVFAASPK
VP7 PO-13    --S------- --------A- -----A---- ----IF--A- -AV---V---

                  60         70         80         90        100
VP7 288356   CFAQNYGIDA PIIGSLDVTI PNKTNDQIGL VSSLCIYYPN EAETEINDTE
VP7 PO-13    --------NV --T-----AV ----D----- T--------- --------N-
                                                             A
                 110        120        130        140        150
VP7 288356   WKSTVAQLLL TKGWPTTSVY LNGYVDLQSF SNNPQLNCDY NIVLIKYNQN
VP7 PO-13    --D------- ---------- ----A----- ---------- ----V--D--

                 160        170        180        190        200
VP7 288356   AGLDISELAE LLLYEWLCNE MDVRLYYYQQ TSEANKWIAM GRDCTIKVCP
VP7 PO-13    ----M----- ---------- ---N------ -A-----L-- -S--------
              B
                 210        220        230        240        250
VP7 288356   LNTQTLGIGC QTTNVATFEQ LTANEKLAII DVVDGVNHKI NYSVASCTIK
VP7 PO-13    ---------- ---D------ ---T------ ---------V --TI-T—-L-
                              C
                 260        270        280        290        300
VP7 288356   NCIRLNQREN VAIIQVGGPE IIDVSEDPMV VPKMIRATRI NWKKWWQVFY
VP7 PO-13    ---------- ---------- ---------I ---------- ----------

                 310        320
VP7 288356   TVIDYINTII QAMSKRSRSL NASAYFLRV
VP7 PO-13    --V------- ---------- -T-T----I
```

Technical Appendix Figure 4. Alignment of the deduced amino acid sequence of VP7 from avian-like RV 288356 and avian rotavirus PO-13. Three potential N-linked glycosylation sites, labeled in red, are present in the Fox-288356 and PO-13 VP7 sequence. Only aa positions 72 and 321 were mapped to hydrophilic regions in Kyte and Doolittle hydropathy plots. Antigenic regions A, B and C, involved in the determination of VP7 serotypes, are underscored. Within these three regions, Fox-288356 rotavirus differed from PO-13 in 7 aa. Identical residues are indicated by dashes.

A

```
                    210        220        230        240        250
VP4 288356   CNYYIIPKNQ TQQLEGFLKN GLPPIQESRY IMPVERLVQN IYQAKPNEDI
VP4 PO-13    -----V--S- -----D---- ---------- ------S--- --R-------
                                           *          §    §
```

B

```
                    310        320
VP4 288356   YERDGETVVA HTTCSVAGVN
VP4 PO-13    ---------- --------I-
```

Technical Appendix Figure 5. Alignment of VP4 amino acid sequences of functional regions from Fox-288356, avian PO-13 and Simian RRV rotavirus. (A) Two of the three arginine residues, important for the VP4 cleavage by tripsin in VP8 and VP5, are conserved in the PO-13 strain (amino acids 229 and 243 in PO-13 strain corresponding to 231 and 247 in RRV, respectively), and only one arginine is conserved in the amino acid sequence of Fox-288356 VP4 (amino acids in position 229), compared to mammalian rotaviruses. Identical residues are indicated by dashes. Arginine residues are indicated by asterisk in the trypsin cleavage regions and different residues are indicated by §. (B) The integrin ligand sequence motif (DGE) is conserved in avian rotavirus PO-13 and in Fox-288356 rotavirus as well as in most of the mammalian rotaviruses.

```
                  10        20        30        40        50
NSP4-288356 MENATTINET L.VEEMYNLT MNYFEHNVIL IKYFPFLASI LTIAFTAWKM
NSP4-PO13   ---------- -.---V--M- -S-------I M--------- ----------
NSP4-Wa/Hu  –DKLADL-Y- -S-ITSM-D- LHSIIQD.PG MA--LYI--V --VL--LH-A
                          H1                          H2
                                                         A
                  60        70        80        90       100
NSP4-288356 GKSTFKITKS VAGSGFKVVR VAIITTFNCI MKTFGSKTEI VSDDRIDVLA
NSP4-PO13   ------V—-T -------I-- -VV—I----V -RL------V -----L-A--
NSP4-Wa/HU  SIP-M-IALK TSKCSY-VIK YC-V-II-TL L-LA-Y-EQV TTK-E-EQQM
                                    H3
                                                  B
                  110       120       130       140       150
NSP4-288356 TRILTEIDRQ VKVIEQLTKR ELEQVKLLAD IYELLKFK.K DGIDMSFETN
NSP4-PO13   SK—-AQ--N- ---------- ---------- ---M----.- -E--------
NSP4-Wa/HU  D--VK-MR-- LEM-DK—--- -I---E—KR- -HDN-ITRPV -V----K-F-
                                  DIR              ISVD
                             C
                  160       170
NSP4-288356 KK...AYEQW I..QDPYKPT RAVSLD
NSP4-PO13   --...E--K- V..K---Q-- ------
NSP4-Wa/HU  Q-NIKTLDE- ESGKN--E-S EVTASM
```

Technical Appendix Figure 6. Alignment of the NSP4 amino acid sequences encoded by Fox-288356, PO-13 and Human Wa strain rotaviruses. Two potential *N*-glycosylation sites are present at the same position as those of mammalian rotaviruses at position 8 and 17, and an additional third potential *N*-glycosylation site is present at amino acid position 3. Only two hydrophobic domains, corresponding to H2 (amino acids 27–47) and H3 (amino acids 67–85), out of the three present in mammalian rotaviruses, are present in Fox-288356 and PO13 strain. These two hydrophobic domains are not identical in Fox-288356 rotavirus compared to PO13 rotavirus: H2 shows two conservative aa exchanges at positions 29-30, H3 shows 5 conservative (position 67 -71 -72 -79 -81) and 2 non conservative (position 75 -82) aa exchanges. Among the five putative functional domains mapped on NSP4, the VP4-binding domain, in particular the diarrhea-inducing region (DIR), is conserved among Fox-288356, PO-13 and mammalian rotavirus; the InterSpecies Variable Domain (ISVD), between residues 131 and 140, exhibits a high degree of sequence variation and mutations, in particular at aa 131, 135 and 138, that are supposed to affect the virulence of the virus as well as the diarrhea-inducing and double-layered particle binding activities of the protein (*9*–*11*). Moreover, the ISVD of the Fox-288356 strain shows a non conservative substitution in aa 140 (140-Gly to Glu), compared to PO13 rotavirus. The oligomerization-associated domain (A), VP4-binding domain (B) and ss particle-binding domain are boxed. The hydrophobic domains are indicated as H1, H2 and H3. The potential *N*-glycosylation sites are in red. The Diarrhea-Inducing Region (DIR) is underlined. The InterSpecies-Variable Domain (ISVD) is in blue. Identical residues are indicated by dashes, conservative substitutions are indicated in bold and gaps are indicated by dots. When analyzing the inferred aa sequence in detail, the NSP4 of the fox isolate was six residues shorter than that of mammalian RVAs.
